# Supplementary material for: Selection of ethanol tolerant strains of Candida albicans by repeated ethanol exposure results in strains with reduced susceptibility to fluconazole
Source: PLoS One. 2024 Feb 20;19(2):e0298724. doi: 10.1371/journal.pone.0298724 (PMC10878505; doi:10.1371/journal.pone.0298724)
Supplement: S14 Table — (DOCX) [file pone.0298724.s023.docx]

**Table S14: Strains Used in This Study**

| **Strain** | **Description** | **Genotype** | **Source** |
| --- | --- | --- | --- |
| SC5314 | Prototroph, clinical Isolate |  | Fonzi and Irwin, 1993. |
| Esc6 | SC5314 background | Chr1 trisomy | This study |
| Esc7 | SC5314 background | Chr3 trisomy, ChrR aneuploid | This study |
| Esc8 | SC5314 background | Chr5 monosomy, Chr6 Trisomy, Chr7 Trisomy, ChrR Trisomy. | This study |
| SN152 | Parent strain | SN152 *arg4∆/arg4, leu2∆/leu2∆, his1∆/his1∆, URA3/ura3∆, IRO1/iro1∆* | Homann et al, 2009 |
| TYE7 | *TYE7* homozygous deletion | SN152 *tye7∆*::*CmLEU2/tye7∆/CdHIS1* | Homann et al, 2009 |
| ACE2 | *ACE2* homozygous deletion | SN152 *ace2∆*::*CmLEU2/ace2∆/CdHIS1* | Homann et al, 2009 |
| TRY4 | *TRY4* homozygous deletion | SN152 *try4∆*::*CmLEU2/try4∆/CdHIS1* | Homann et al, 2009 |
| SKO1 | *SKO1* homozygous deletion | SN152 *sko1∆*::*CmLEU2/sko1∆/CdHIS1* | Homann et al, 2009 |
| SFL2 | *SFL2* homozygous deletion | SN152 *sfl2∆*::*CmLEU2/sfl2∆/CdHIS1* | Homann et al, 2009 |
| YOX1 | *YOX1* homozygous deletion | SN152 *yox1∆*::*CmLEU2/yox1∆/CdHIS1* | Homann et al, 2009 |
| ARG81 | *ARG81* homozygous deletion | SN152 *arg81∆*::*CmLEU2/arg81∆/CdHIS1* | Homann et al, 2009 |
| ZCF19 | *ZCF19* homozygous deletion | SN152 *zcf19∆*::*CmLEU2/zcf19∆/CdHIS1* | Homann et al, 2009 |
| MRR1 | *MRR1* homozygous deletion | SN152 *mrr1∆*::*CmLEU2/mrr1∆/CdHIS1* | Homann et al, 2009 |
| MRR2 | *MRR2* homozygous deletion | SN152 *mrr2∆*::*CmLEU2/mrr2∆/CdHIS1* | Homann et al, 2009 |
| NDT80 | *NDT80* homozygous deletion | SN152 *ndt80∆*::*CmLEU2/ndt80∆/CdHIS1* | Homann et al, 2009 |
| UPC2 | *UPC2* homozygous deletion | SN152 *upc2∆*::*CmLEU2/upc2∆/CdHIS1* | Homann et al, 2009 |
| CAP1 | *CAP1* homozygous deletion | SN152 *cap1∆*::*CmLEU2/cap1∆/CdHIS1* | Homann et al, 2009 |
| CRZ1 | *CRZ1* homozygous deletion | SN152 *crz1∆*::*CmLEU2/crz1∆/CdHIS1* | Homann et al, 2009 |
| MSN4 | *MSN4* homozygous deletion | SN152 *msn4∆*::*CmLEU2/msn4∆/CdHIS1* | Homann et al, 2009 |
| MNL1 | *MNL1* homozygous deletion | SN152 *mnl1∆*::*CmLEU2/mnl1∆/CdHIS1* | Homann et al, 2009 |
| GZF3 | *GZF3* homozygous deletion | SN152 *gzf3∆*::*CmLEU2/gzf3∆/CdHIS1* | Homann et al, 2009 |
| SSN6 | *SSN6* homozygous deletion | SN152 *ssn6∆*::*CmLEU2/ssn6∆/CdHIS1* | Homann et al, 2009 |
| UPC2-G648D | *UPC2* mutant | *SC5314 UPC2-G648D::FRT/UPC2-G648D::FRT* | Flowers et al, 2012 |
|  |  |  |  |
