# Supplementary material for: Selection of ethanol tolerant strains of Candida albicans by repeated ethanol exposure results in strains with reduced susceptibility to fluconazole
Source: PLoS One. 2024 Feb 20;19(2):e0298724. doi: 10.1371/journal.pone.0298724 (PMC10878505; doi:10.1371/journal.pone.0298724)
Supplement: S15 Table — (DOCX) [file pone.0298724.s024.docx]

| **Primer Name For qPCR** | **Sequence** | **Source** |
| --- | --- | --- |
| ERG11-FW | GCTAATTCTGTTTCATTTAACTCTTCTGAT | [1] |
| ERG11-RV | GGACCAGCTTCGGTATCCAAA |  |
| ERG2-FW | CAGCAATTGGGACTGAAGGT | [2] |
| ERG2-RV | TTCGGGAATCAATGCACCAG |  |
| ERG4-FW | CTTCGGAAGGTCAATCTTGG | [2] |
| ERG4-RV | GTCCAAACACCGGGTAAAGT |  |
| ERG5-FW | GAAGAGCAATTGCGTGTGAG | [2] |
| ERG5-RV | TGGTGGACGGTATCTCAAAG |  |
| ERG6-FW | AGATGCTGCTTCTGTTGCTG | [2] |
| ERG6-RV | GGAATGAAGAACCCCAACC |  |
| ERG25-FW | TATTTCATTGGTGGATACTCTTCATCTT | [1] |
| ERG25-RV | GGACCAGCTTCGGTATCCAAA |  |
| TPS1-FW | TCGCAAGGGTGTCTTGATCTTAT | [3] |
| TPS1-RV | AACAATCAAGGCACCATTAAGTGA |  |
| TPS2-FW | TTGCTGTTGGTCCTGCATCA | [3] |
| TPS2-RV | GGCGAGGTTCGTTCAAATGT |  |
| CDR1-FW | GGTCAACTTGTAATGGGTC | [4] |
| CDR1-RV | AGGACGATAAAGGGCATA |  |
| HSP90-FW | GGGAATCTAACGCTGGTGGTAA | [5] |
| HSP90-RV | TTCGGTTTCTGGAACTTCTTTT |  |
| ADH1-FW | CACTCACGATGGTTCATTCG | [6] |
| ADH1-RV | AAGATGGTGCGACATTGG |  |
| ADH2-FW | AAATGGTTGAACGGCTCTTG | [7] |
| ADH2-RV | GACGGTGACACCAGCACATAAG |  |
| ADH3-FW | ATTCCGACAAATACATTAAAATTAGAGG | This Study: Made with NCBI Primer Blast (Target Validated) |
| ADH3-RV | AATAACCACCAAAATTGAAAATAACTT |  |
| ADH4-FW | TACTGATTCTTATGGATTATATCAAGGA | This Study: Made with NCBI Primer Blast (Target Validated) |
| ADH4-RV | AAAAATCTAACAATTGGAACAATATCAG |  |
| ADH5-FW | ACCTGCAAGGGCTCATTCTG | [8] |
| ADH5-RV | CGGCTCTCAACTTCTCCATA |  |
| ALD4-FW | TTATGCCGTTGAATGTGCTC | [7] |
| ALD4-RV | CTTTGCCCGTGATTTTATCAGC |  |
| ALD5-FW | TGTTGTTACCGGTGGTGCTA | [7] |
| ALD5-RV | CAACGGCTTCGTCAACAGTA |  |
| ACS1-FW | ATTTGCCAGCTTGGTTCATC | [7] |
| ACS1-RV | CACCCTTTTTAACCCCCAAT |  |
| ACS2-FW | CTCAAGGATTTTTCGGTCCA | [7] |
| ACS2-RV | ATTCACCACCCAAAAACCAA |  |
| MDR1-FW | ACATAAATACTTTGCCCATCCAGAA | [9] |
| MDR1-RV | AAGAGTTGGTTTGTAATCGGCTAAA |  |
| SPL1-FW | AAAGGATACTGTGTTAGTTTCTATTATG | This Study: Made with NCBI Primer Blast (Target Validated) |
| SPL1-RV | TATTTTCACGACATATTTTACCAATTTC |  |
| ACT1-FW | GTTGGTGATGAAGCCCAATC | [10] |
| ACT1-RV | CCCAGTTGGAAACAATACCG |  |
| CDR2-FW | GCCAATGCTGAACCGACA | [4] |
| CDR2-RV | ACCAGCCAATACCCCACA |  |

**Supplemental Bibliography**

1. Nailis H, Vandenbosch D, Deforce D, Nelis HJ, Coenye T. Transcriptional response to fluconazole and amphotericin B in Candida albicans biofilms. Res Microbiol. 2010 May;161(4):284–92.

2. Su H, Han L, Ding N, Guan P, Hu C, Huang X. Bafilomycin C1 exert antifungal effect through disturbing sterol biosynthesis in Candida albicans. J Antibiot (Tokyo). 2018 Apr;71(4):467–76.

3. Guirao-Abad JP, Sánchez-Fresneda R, Román E, Pla J, Argüelles JC, Alonso-Monge R. The MAPK Hog1 mediates the response to amphotericin B in Candida albicans. Fungal Genet Biol. 2020 Mar;136:103302.

4. Li WJ, Liu JY, Shi C, Zhao Y, Meng L ning, Wu F, et al. FLO8 deletion leads to azole resistance by upregulating CDR1 and CDR2 in Candida albicans. Res Microbiol. 2019 Sep;170(6–7):272–9.

5. Dai B, Wang Y, Li D, Xu Y, Liang R, Zhao L, et al. Hsp90 Is Involved in Apoptosis of Candida albicans by Regulating the Calcineurin-Caspase Apoptotic Pathway. Nielsen K, editor. PLoS ONE. 2012 Sep 18;7(9):e45109.

6. Bakri MM, Rich AM, Cannon RD, Holmes AR. *In vitro* expression of *Candida albicans* alcohol dehydrogenase genes involved in acetaldehyde metabolism. Mol Oral Microbiol. 2015 Feb;30(1):27–38.

7. Nieminen MT, Novak-Frazer L, Rautemaa V, Rajendran R, Sorsa T, Ramage G, et al. A Novel Antifungal Is Active against Candida albicans Biofilms and Inhibits Mutagenic Acetaldehyde Production In Vitro. Bassilana M, editor. PLoS ONE. 2014 May 27;9(5):e97864.

8. Gonçalves B, Bernardo R, Wang C, Schröder MS, Pedro NA, Butler G, et al. Effect of progesterone on Candida albicans biofilm formation under acidic conditions: A transcriptomic analysis. Int J Med Microbiol. 2020 Apr;310(3):151414.

9. Thomas E, Roman E, Claypool S, Manzoor N, Pla J, Panwar SL. Mitochondria Influence *CDR1* Efflux Pump Activity, Hog1-Mediated Oxidative Stress Pathway, Iron Homeostasis, and Ergosterol Levels in Candida albicans. Antimicrob Agents Chemother. 2013 Nov;57(11):5580–99.

10. Junier A, Weeks A, Alcaraz Y, Kumamoto CA. Bypass of Dfi1 Regulation of Candida albicans Invasive Filamentation by Iron Limitation. Mitchell AP, editor. mSphere. 2022 Feb 23;7(1):e00779-21.

11. Fonzi WA, Irwin MY. Isogenic strain construction and gene mapping in Candida albicans. Genetics. 1993 Jul 1;134(3):717–28.

12. Homann OR, Dea J, Noble SM, Johnson AD. A Phenotypic Profile of the Candida albicans Regulatory Network. Copenhaver GP, editor. PLoS Genet. 2009 Dec 24;5(12):e1000783.

13. Flowers SA, Barker KS, Berkow EL, Toner G, Chadwick SG, Gygax SE, et al. Gain-of-Function Mutations in *UPC2* Are a Frequent Cause of *ERG11* Upregulation in Azole-Resistant Clinical Isolates of Candida albicans. Eukaryot Cell. 2012 Oct;11(10):1289–99.
